# Vaccination status as a determinant of hospitalization in influenza: Insights from emergency department data

**DOI:** 10.1007/s10096-025-05401-4

**Published:** 2026-01-08

**Authors:** Merve Saracoglu Sumbul, Mehmet Can Erisen, Berçem Berent Kaya, Hilmi Erdem Sumbul, Ramazan Azim Okyay, Burhan Fatih Kocyigit

**Affiliations:** 1 Sarıçam District Health Directorate, Family Medicine Department, Adana, Türkiye; 2 Department of Internal Medicine, University of Health Sciences, Adana Health Practice and Research Center, Adana, Türkiye; 3 Department of Public Health, Adana Faculty of Medicine, University of Health Sciences, Adana, Türkiye; 4 Department of Physical Medicine and Rehabilitation, University of Health Sciences, Adana City Research and Training Hospital, Adana, Türkiye

**Keywords:** Influenza, Influenza vaccines, Vaccination, Hospitalization

## Abstract

**Purpose:**

This study aims to investigate the effect of seasonal influenza vaccination on hospitalization rates among patients presenting to the emergency department with influenza-like illness.

**Methods:**

A retrospective, single-center observational study was conducted, involving adult patients with influenza (ICD-10 codes J10 and J11) diagnosed in the emergency department between May 2024 and April 2025. Clinical and demographic information was collected from electronic records, and vaccination status was confirmed through follow-up phone calls. To tackle the “zero event” problem—no hospitalizations among vaccinated individuals—advanced statistical modeling was employed, including standard logistic regression, and Bayesian logistic regression using Markov Chain Monte Carlo (MCMC) simulations. Odds ratios (OR) and 95% Highest Density Intervals (HDI) were calculated to assess the effectiveness of vaccination.

**Results:**

A total of 878 patients were enrolled: 3.3% (*n* = 29) received vaccinations, while 2.7% (*n* = 24) required hospitalization. None of the vaccine recipients were hospitalized. Standard logistic regression indicated that age was a significant indicator of hospitalization. Furthermore, Bayesian logistic regression followed, which confirmed vaccination’s statistically significant protective effect. The OR for vaccination was 0.526 (95% HDI: 0.336–0.739), indicating a 47% reduction in hospitalization risk among vaccinated individuals.

**Conclusion:**

Seasonal influenza vaccination was significantly associated with a lower risk of hospitalization in patients presenting with influenza-like illness to the emergency department. These findings support public health initiatives to enhance influenza vaccine coverage, particularly for the elderly.

**Supplementary Information:**

The online version contains supplementary material available at 10.1007/s10096-025-05401-4.

## Introduction

Influenza is a common acute respiratory infection that causes significant global morbidity and mortality, with the World Health Organization estimating 3–5 million severe cases and up to 650,000 respiratory fatalities annually [1, 2]. While the majority of patients typically recover within a few days, influenza has the potential to lead to severe complications, particularly among at-risk populations such as the elderly, individuals with chronic diseases, and those with compromised immune systems [3].

Annual influenza vaccination is the most effective preventive strategy [4], consistently proven to reduce illness severity, hospitalizations, and mortality [5–7]. Despite these advantages, influenza vaccination rates remain insufficient in certain countries due to vaccine hesitancy, inadequate awareness, or limited vaccination access [8, 9].

Seasonal influenza outbreaks lead to a considerable increase in emergency department visits and hospital admissions [10]. These events substantially burden healthcare organizations, affecting both resources and financial costs. Therefore, reducing hospitalizations through vaccination is essential for individual health, public health, and economic stability [11].

In light of this context, assessing the real-world effectiveness of the influenza vaccine in hospital admissions is of significant clinical importance. Observational data from emergency departments can provide valuable insights into disease burden and the preventive effectiveness of immunization. This study aims to evaluate the influence of seasonal influenza vaccination on the likelihood of hospitalization in patients with influenza-like illness who attend the emergency department. We employed classical and advanced statistical methods to derive a reliable estimate of the vaccine’s protective effect in a real-world clinical environment.

## Method

### Study design

This is a retrospective cohort study. The study population consisted of patients who presented to the emergency department of Adana City Research and Training Hospital in May 2024 - April 2025 with complaints of influenza-like illness and were diagnosed with J10 (influenza with identified virus) and J11 (influenza without identified virus) according to ICD-10 codes. Clinical and demographic data of the patients were obtained from electronic medical records. Vaccination status information was collected by phone calls to the patients, as this data was not consistently available in the electronic medical records.

## Inclusion and exclusion criteria

Patients were eligible if they:

- Were aged 18 years or older.
- Presented to the emergency department between May 2024 and April 2025 with influenza-like illness.
- Received a diagnosis of influenza according to ICD-10 codes J10 (influenza with identified virus) or J11 (influenza without identified virus).

Patients with duplicate medical records were excluded. No other exclusion criteria were applied to ensure comprehensive representation of all influenza cases during the study period.

### Patient selection process

**Fig. 1:**
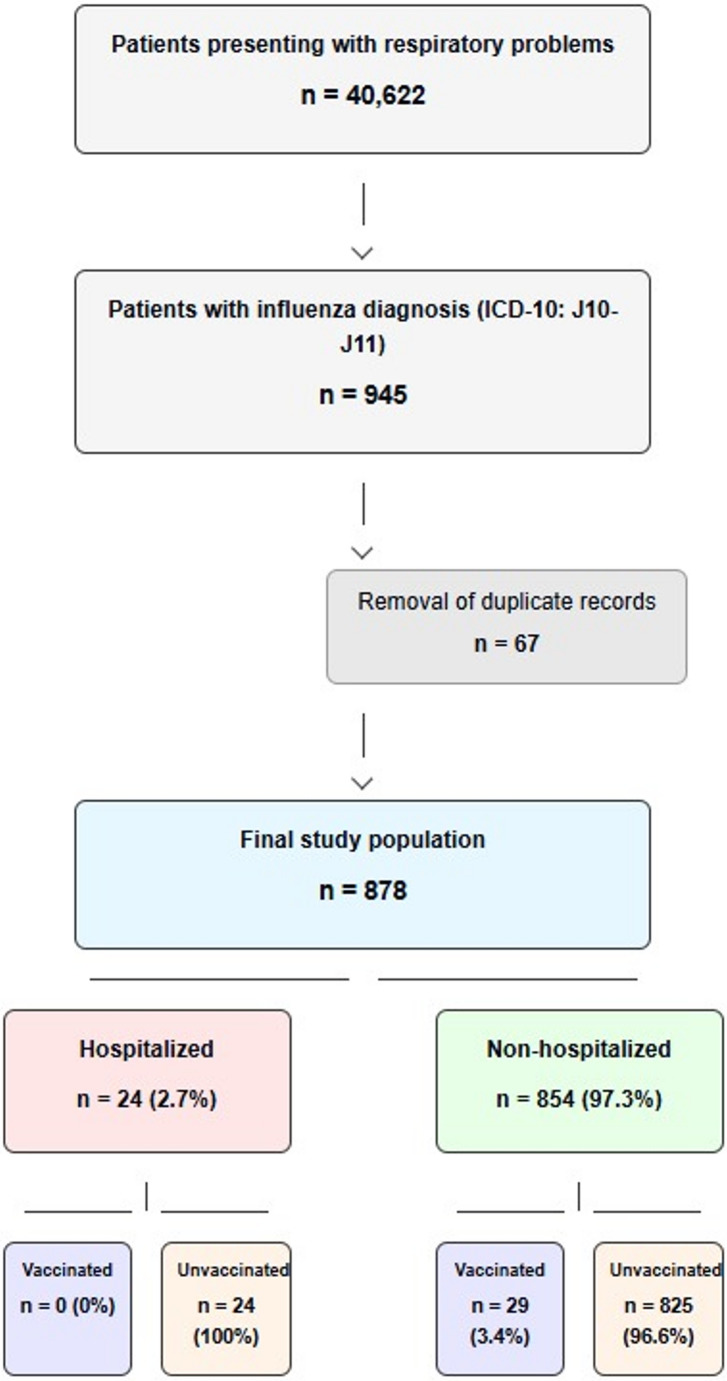
Patient selection process

The patient selection process began with a review of 40,622 patients who presented to the emergency department with respiratory problems. Of these, 945 patients were diagnosed with influenza (ICD-10 codes J10 and J11). After excluding duplicate records (*n* = 67), the final study population consisted of 878 patients (Fig. 1).

### Charlson comorbidity index

In our study, the Charlson Comorbidity Index (CCI) was used to assess the comorbidity burden of the participants objectively. The CCI is a scoring system that assigns a weighted score to specific chronic diseases an individual has, and is widely used to predict mortality risk. Each comorbid condition in this index is evaluated with a specific score according to the effect of the disease on prognosis, and the total score reflects the individual’s comorbidity burden [12, 13].

### Exposure (Potential risk factors)

The primary exposure variable was influenza vaccination status (vaccinated vs. unvaccinated). Additional potential risk factors evaluated in the investigation comprised age (continuous), sex (male/female), and the CCI score, which measures the burden of comorbidities.

### Outcome measure

The primary outcome was whether or not the patient was hospitalized after visiting the emergency department for influenza-like illness. Hospitalization was defined as being admitted to the hospital directly from the emergency department during the initial visit.

## Statistical analyses

The Python programming language (version 3.9) was used for data analysis. Statistical analyses were performed using the following Python libraries: pandas (for data manipulation), numpy (for numerical operations), scipy. stats (for basic statistical tests) and statsmodels (for statistical modeling).

The conformity of continuous variables to a normal distribution was assessed using the test_shapiro_wilk() function. The mann_whitney_u_test() function was used to compare two groups of data that did not fit the normal distribution. The chi_square_independence() function was used to compare categorical variables.

A logistic regression model was established to predict the factors affecting hospitalization using the sm. Logit and sm. GLM modules of the statsmodels library. In the first analysis, all variables (age, sex, CCI, and vaccination status) were included. However, the odds ratio values for vaccination approached infinity in standard logistic regression because of the absence of hospitalization in vaccinated individuals (the “zero event” problem). Therefore, in the first stage, only age, sex, and CCI variables were included in the first logistic regression model, which was presented in Figs. 5 and 6.

To overcome the methodological problem and put the vaccination status in a regression model, further analysis methods were applied in our study:

Bayesian Logistic Regression: Bayesian logistic regression analysis based on Markov Chain Monte Carlo (MCMC) simulations was performed on the data using the PyMC library (version PyMC 5.22.0). The No U-Turn Sampler (NUTS) algorithm was used for MCMC sampling (2000 draws, 1000 tune/burn-in iterations, target_accept = 0.9, cores = 1, random_seed = 42), and the results were analyzed with the ArviZ library (version 0.21.0).

We used specific priors for parameter estimates:

- Intercept: A weakly non-informative prior, Normal(0, 10), was used.
- Vaccination Status: An informative prior, Normal(−0.64, 0.2), was used. This prior was chosen based on previous studies suggesting that vaccination can reduce hospitalization risk by approximately 30–60% [14–16]. The log-odds equivalent for this risk reduction (e.g., an odds ratio of 0.5 implies a log-odds of ln(0.5) ≈ −0.69) guided the choice of this prior mean, with a relatively narrow standard deviation reflecting some confidence in this prior information.
- Sex, Age, and CCI: Weakly non-informative priors, Normal(0, 1), were used for these covariates, allowing the data to have a stronger influence on their estimates while still providing some regularization.

For sensitivity analysis, we initially used weakly non-informative priors (Normal(0, 5)) for all parameters, which resulted in an estimated 86% reduction in hospitalization risk among vaccinated patients. However, recognizing that weakly informative priors may lead to overstated effects, we subsequently implemented the more conservative informative prior for vaccination status described above, which better aligns with existing literature while still allowing the data to contribute substantially to the posterior estimates (Appendix).

We calculated 95% Highest Density Intervals (HDI) from posterior distributions and evaluated the convergence and mixing quality of MCMC chains for the reliability of parameter estimates. Odds ratio values were calculated by exponentiating the regression coefficients, and their 95% HDI were determined. Statistical significance was accepted if the 95% HDI the odds ratio did not include a value of 1.

Python scripts were developed using the matplotlib and seaborn libraries for the visualization of results.

### Ethical considerations

The study was approved by the local ethics committee with decision number 480 on 10 April 2025.

## Results

**Fig. 2:**
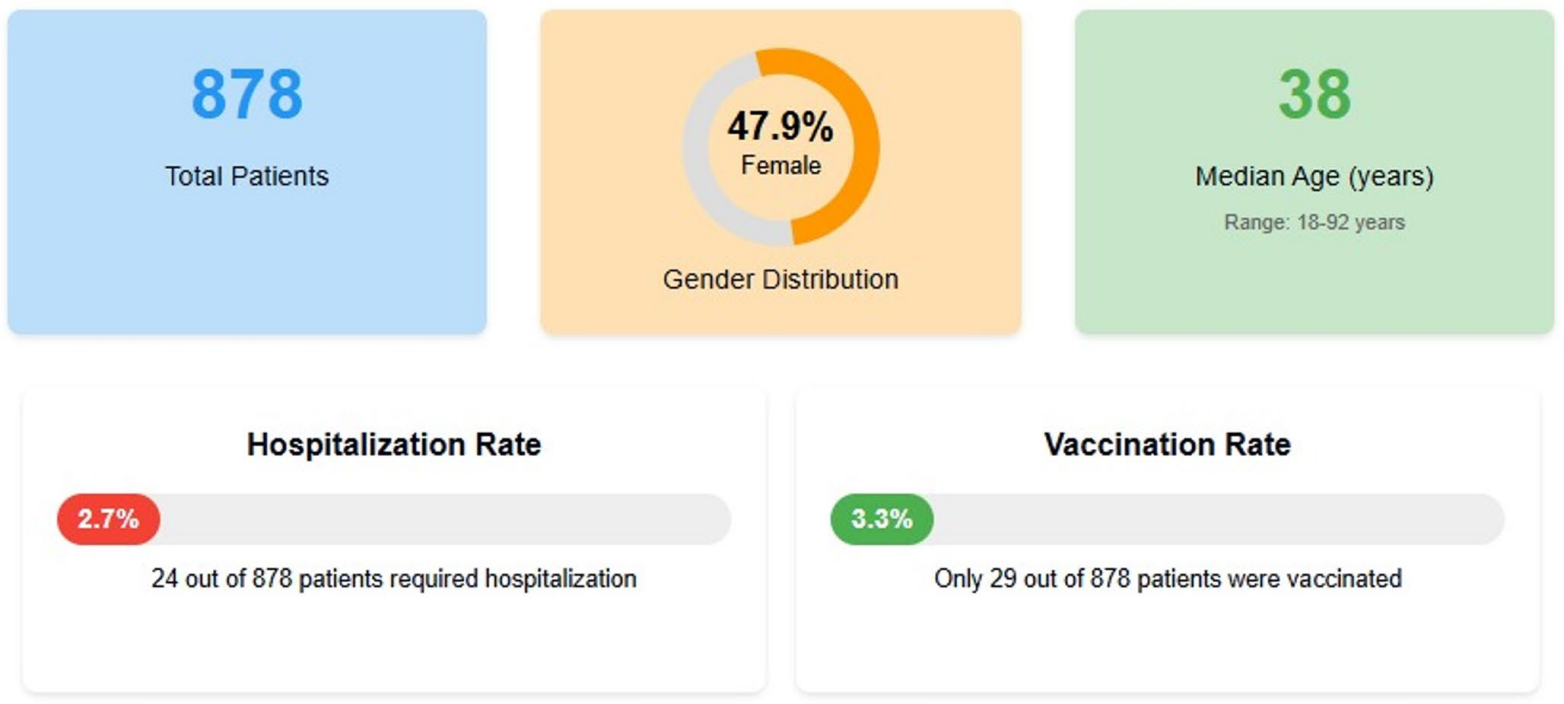
Demographics of patients

A total of 878 patients were included in the study. Of these, 421 were female (47.9%). The median age of the patients was 38.0 (min = 18, max = 92). 24 (2.7%) of the patients were hospitalized. Only 29 patients (3.3%) had received influenza vaccination before their hospital admission (Fig. 2). When the relationship between hospitalization status and sex was evaluated, no statistical difference was found between the groups (χ2 = 0.044, *p* = 0.833).

**Fig. 3:**
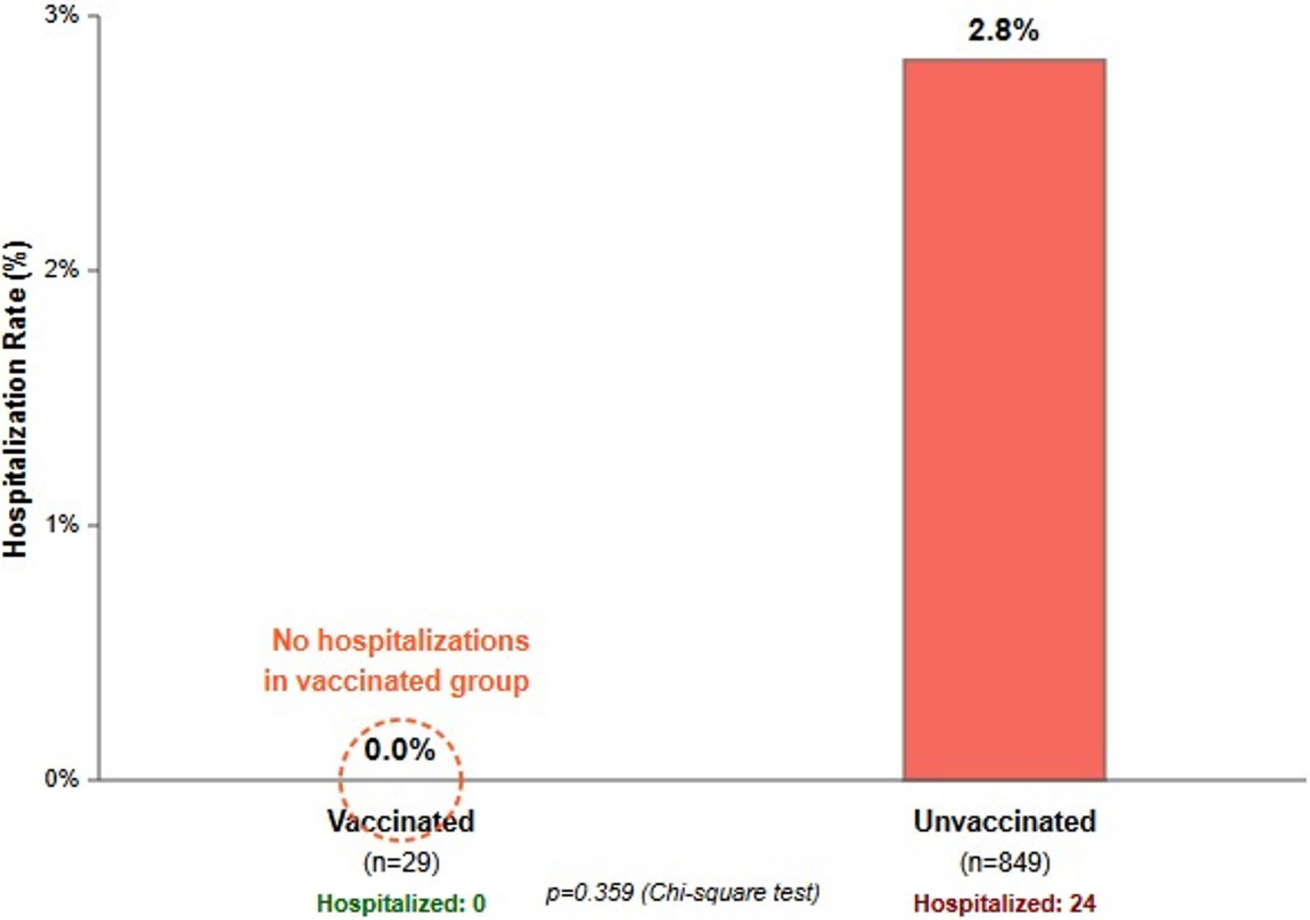
Hospitalization rates by vaccination status *Although the difference between vaccinated and unvaccinated groups was not statistically significant, no hospitalizations were observed in the vaccinated patients

Strikingly, no one who was vaccinated had a hospital stay (0 of 29). However, this difference was not statistically significant (χ2 = 0.843, *p* = 0.359) (Fig. 3).

**Fig. 4:**
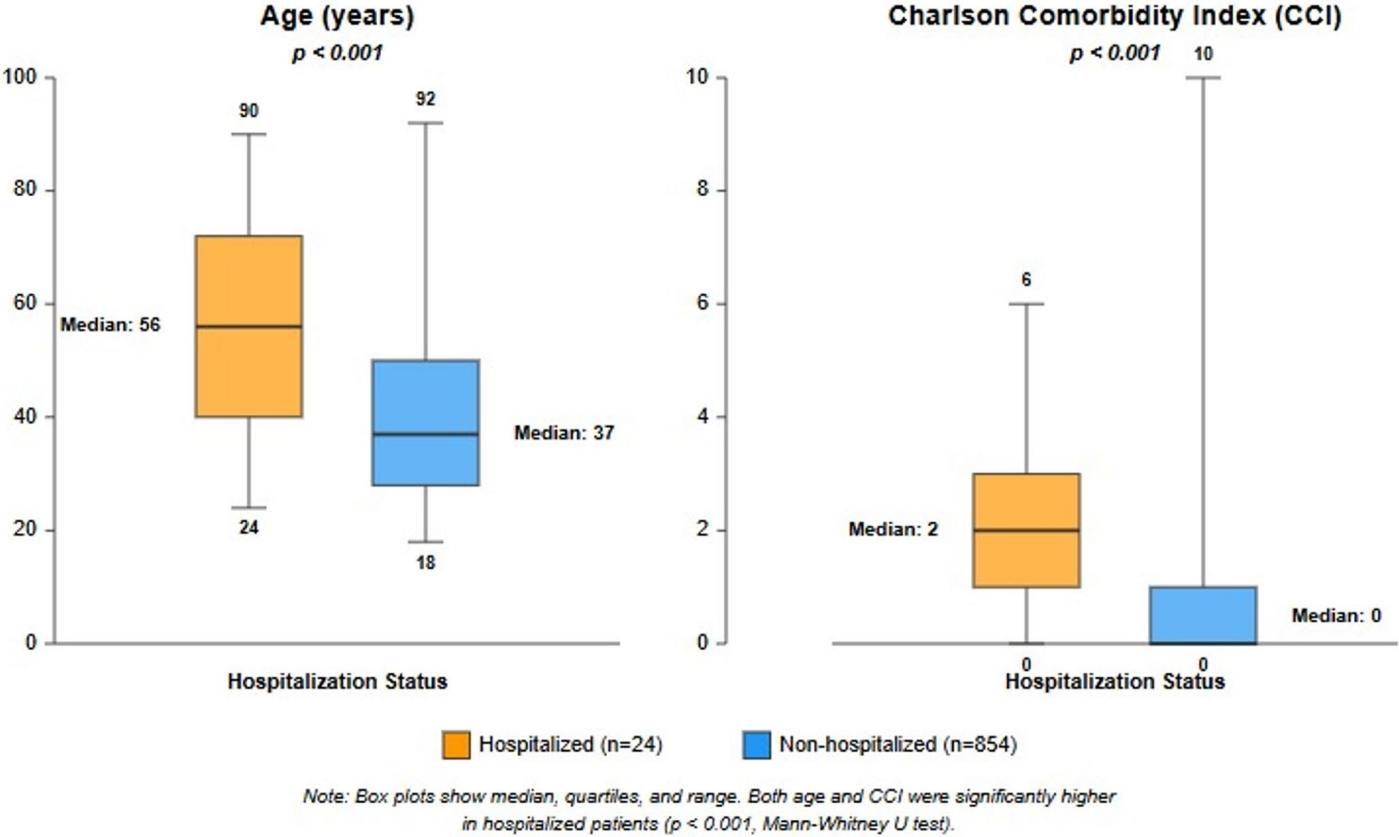
Age and CCI distribution by hospitalization status *Box plots show median, quartiles, and range. Both Age and CCI were significantly higher in hospitalized patients (p<0.001)

In univariate analyses performed between hospitalization and CCI and patient age, it was found that the CCI and age of hospitalized patients were significantly higher than those who were not hospitalized (Fig. 4).

**Fig. 5:**
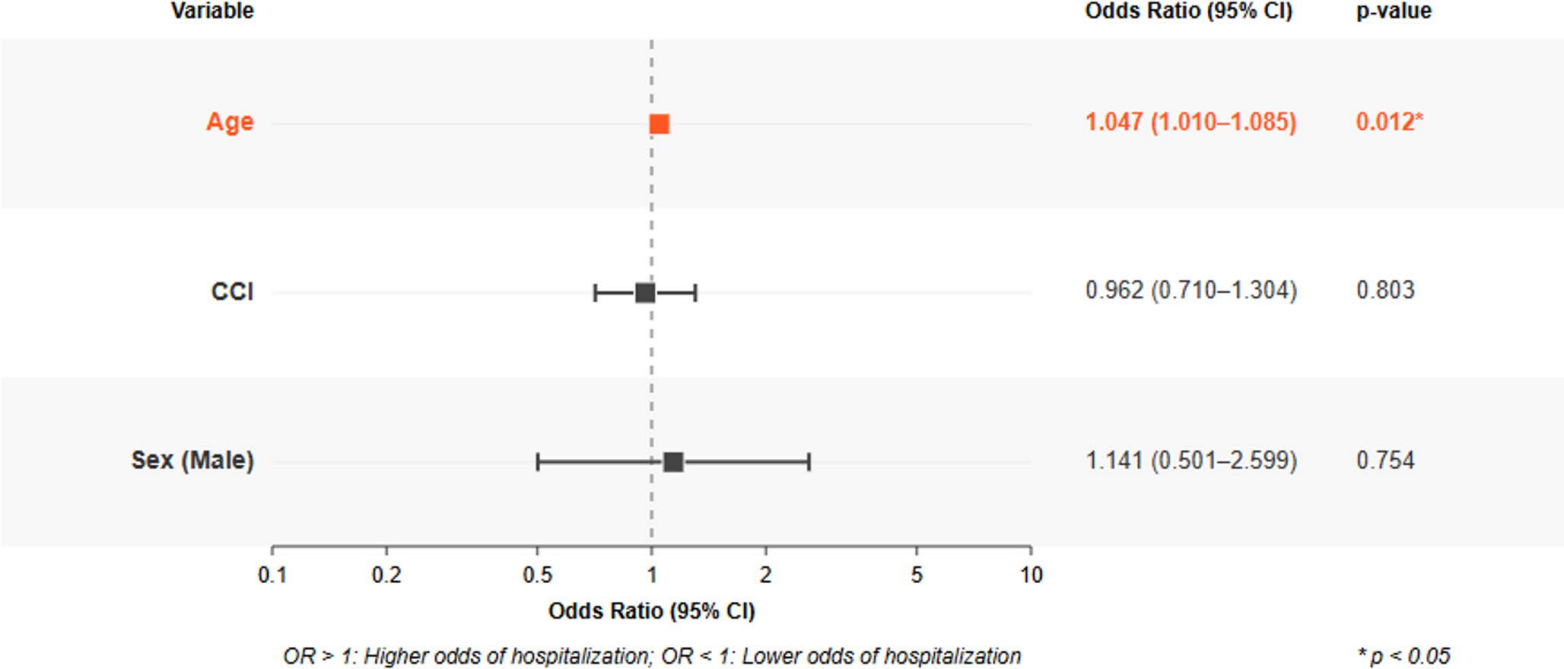
Predictors of hospitalization *Enter method, Dependent variable: hospitalization 0=no, 1=yes. For every 1-year increase in age, the odds of hospitalization increase by 4.7% (OR=1.047, 95% Confidence Interval (CI): 1.010-1.085, p=0.012), indicating that older age significantly increases hospitalization risk. CCI and sex were not significant predictors of hospitalization

**Fig. 6:**
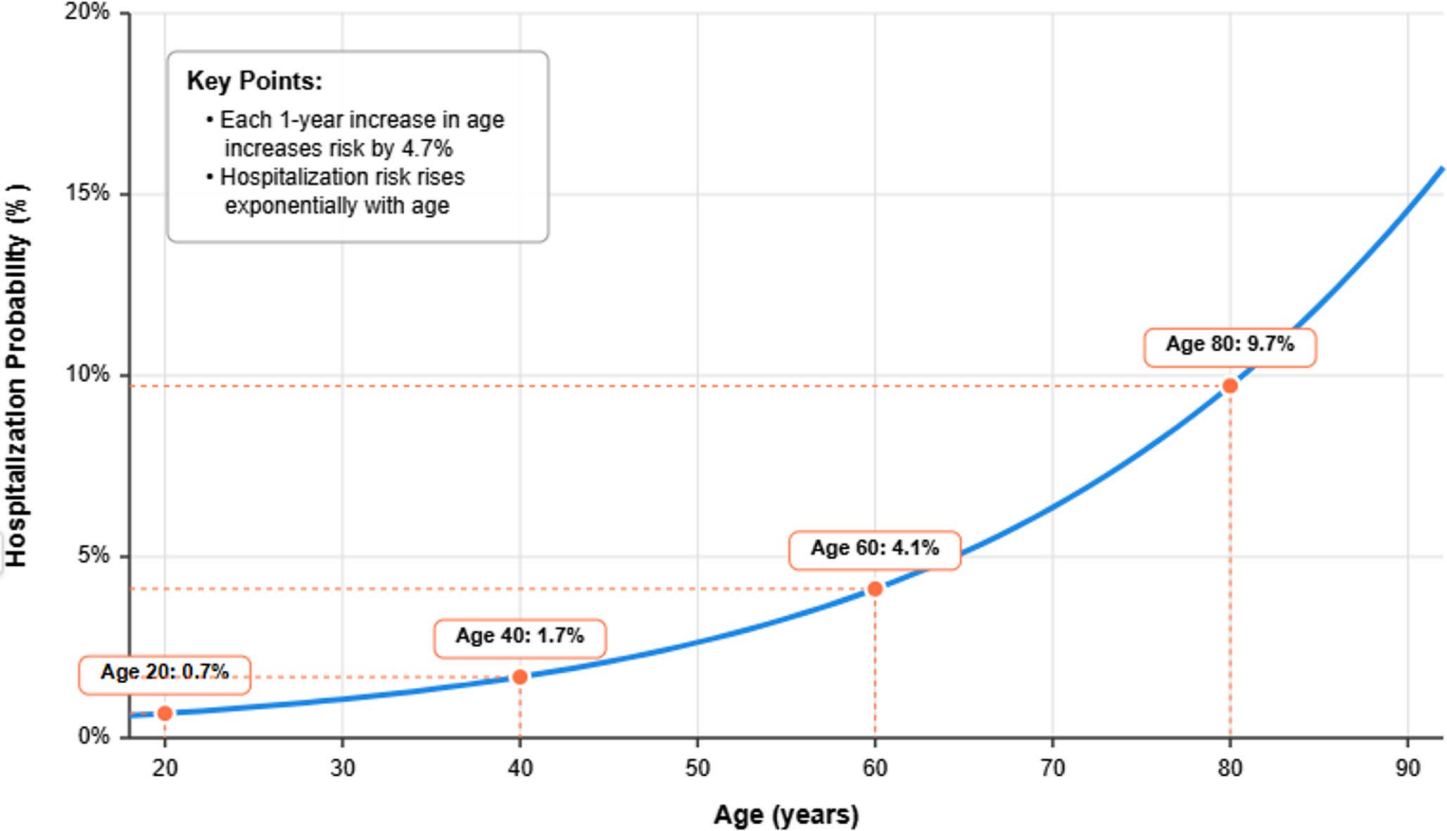
Predicted hospitalization probability by age *This curve shows how the predicted probability of hospitalization increases with age based on the logistic regression model. The model estimates that for each one-year increase in age, the odds of hospitalization increase by 4.7% (OR=1.047, 95% CI: 1.010-1.085, p=0.012). Note that while the absolute risk remains relatively low, there is a clear exponential increase with age

In the regression analysis, only age was found to affect hospitalization (Fig. 5). Each increase in age increased hospitalization by 4.7% (Fig. 6).

**Fig. 7:**
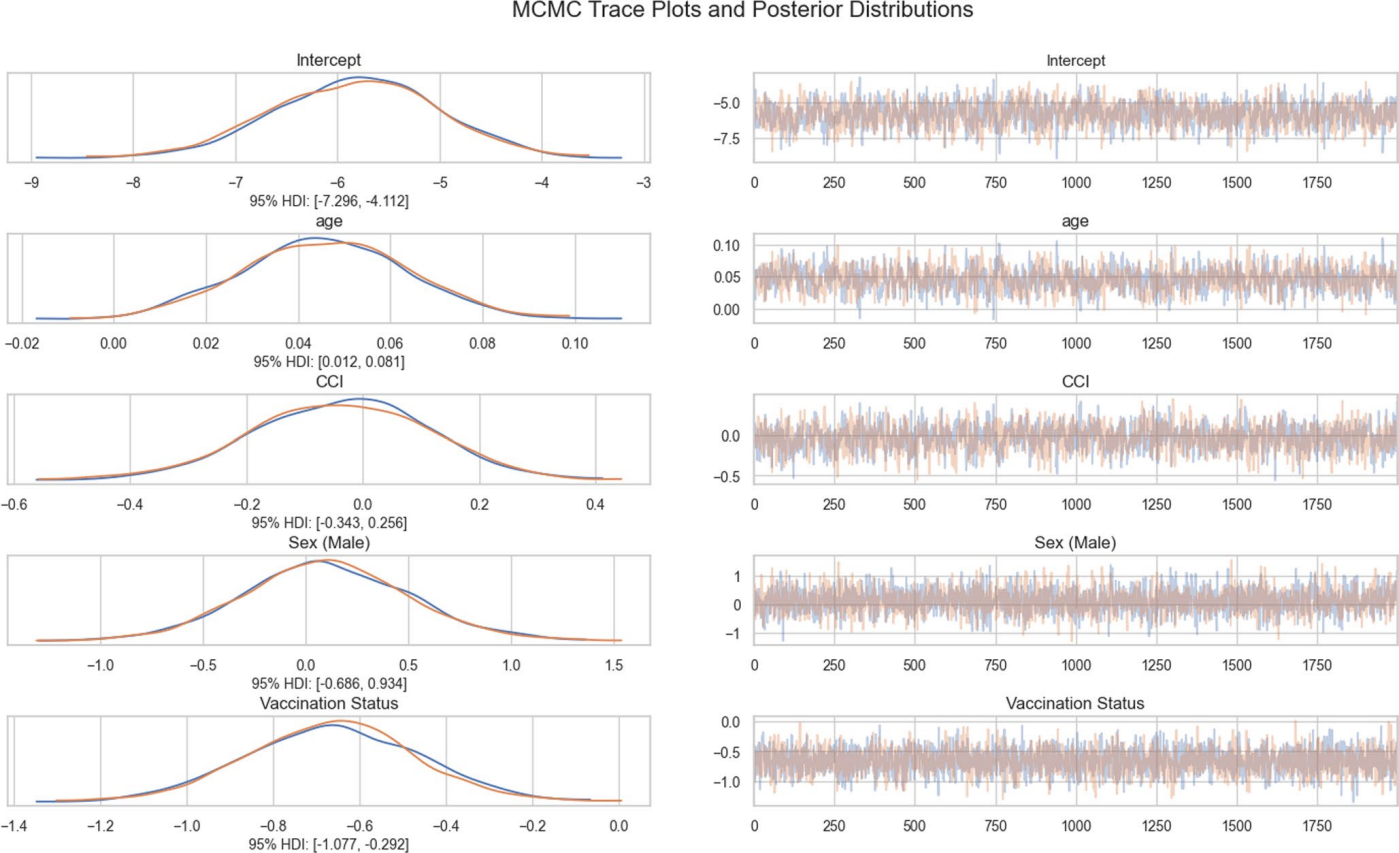
MCMC trace plots and posterior distributions. MCMC trace plots (left panels) and posterior distributions (right panels) for all model parameters from Bayesian logistic regression analysis. Trace plots demonstrate excellent convergence with the characteristic "fuzzy caterpillar" pattern indicating proper mixing of chains across 2000 post-warmup draws. Posterior distributions are smooth and unimodal, confirming convergence. Vaccination and age parameters show statistically significant effects (HDIs do not include zero), while sex and CCI parameters are non-significant

The fact that none of the patients who received influenza vaccination experienced hospitalization (the “zero event” problem) limited the ability to reliably assess the effectiveness of vaccination in standard logistic regression analysis, resulting in excessively high odds ratio values. To overcome this methodological challenge, we applied Bayesian logistic regression analysis. Bayesian logistic regression analysis results revealed a significant effect of vaccination status on hospitalization. When posterior distributions and 95% HDI were evaluated, the HDI obtained for vaccination status was entirely negative [−1.077, −0.292], and the HDI obtained for age was completely positive [0.012, 0.081], indicating that vaccination significantly reduced the risk of hospitalization, whereas increasing age elevated it. On the other hand, since the HDI for sex [−0.686, 0.934] and CCI [−0.343, 0.256] variables included zero, the effects of these variables on hospitalization did not reach statistical significance (Fig. 7).

**Fig. 8:**
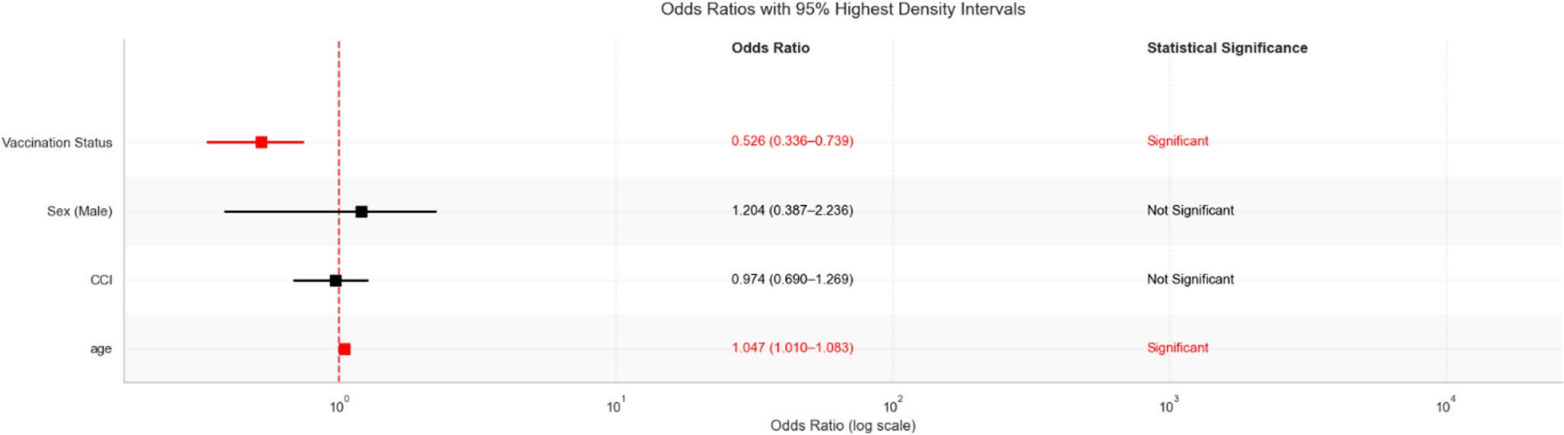
Predictors of hospitalization in Bayesian Regression. Forest plot showing odds ratios with 95% Highest Density Intervals (HDI) from Bayesian logistic regression analysis. Vaccination status demonstrates a significant protective effect (OR = 0.526, 95% HDI: 0.336-0.739), indicating a 47% reduction in hospitalization risk among vaccinated patients. Age shows a significant risk-increasing effect (OR = 1.047, 95% HDI: 1.010-1.083), with each year of age increasing hospitalization odds by 4.7%

Similarly, the odds ratio values obtained demonstrate the protective effect of vaccination on hospitalization. The odds ratio for vaccination was 0.526 (95% HDI: 0.336–0.739), indicating that vaccinated patients had a 47% lower risk of hospitalization than unvaccinated patients, and for age, 1.047 (95% HDI: 1.010–1.083), indicating that the hospitalization rate increases by each year lived. The odds ratio values obtained for the variables sex (OR = 1.204, 95% HDI: 0.387–2.236) and CCI (OR = 0.974, 95% HDI: 0.690–1.269) did not reach statistical significance (Fig. 8).

## Discussion

### Key results

This observational study retrospectively examined 878 patients who visited the emergency department with influenza-like illness, focusing on factors associated with hospitalization and the impact of seasonal influenza vaccination. Among the cohort, 24 patients (2.7%) were hospitalized, and 29 patients (3.3%) received the influenza vaccine before admission. Importantly, none of those vaccinated experienced hospitalization. Both standart logistic regression analysis and Bayesian model.revealed that age is a significant independent predictor of hospitalization, with each additional year of age increasing the risk by approximately 4.7%. Neither sex nor CCI was determined to be a statistically significant variable in any of the analytical models. Bayesian logistic regression demonstrated that vaccination was significantly associated with a decreased risk of hospitalization, showing a 47% reduction in hospitalization risk among vaccinated individuals.

### Hospitalization and vaccination rates

Influenza hospitalization rates fluctuate markedly based on the demographic examined and the therapeutic context. In our examination of emergency department patients presenting with influenza-like illness, the hospitalization rate was 2.7%. Appiah et al. [17] noted a hospitalization rate of 1.7% among 7,813 outpatients with laboratory-confirmed influenza. However, McLean et al. [18] documented higher hospitalization rates of 6% for influenza A and 5% for influenza B cases with hospital visits. These discrepancies presumably reflect variations in demographic variables, disease severity, and medical care trajectories. In contrast to Appiah et al. [17], our marginally elevated rate may be attributed to the emergency department environment, which often attracts more critically ill cases. The reduced rate relative to McLean et al. [18] may arise from case confirmation methodologies or admission criteria variations. Our hospitalization rate aligns with the anticipated range for emergency department-assessed influenza groups, underscoring the significant burden that outpatient-managed influenza can impose on acute care facilities.

In our study, only 3.3% of patients presenting to the emergency department with influenza-like illness indicated that they had received the seasonal influenza vaccine, a figure significantly lower than the 48.6% pooled vaccination rate documented in a comprehensive meta-analysis of outpatient populations by Shehadeh et al. [19]. Hiller et al. [20] discovered that 41.5% of emergency department patients were classified as high-risk, with 43.5% of this group having received vaccinations. Several factors may contribute to this exceedingly low vaccination rate, including vaccine hesitancy, inadequate public awareness initiatives, and systemic obstacles to healthcare access. Regional variations in health-seeking behavior, socioeconomic inequities, and the limited availability of influenza vaccination programs for individuals outside high-risk groups may all contribute to these findings. Furthermore, immunization opportunities during outpatient visits and hospitalizations may be underused, resulting in missed opportunities for preventative care. This highlights the urgent need for targeted public health initiatives to boost vaccination rates and reduce influenza-related hospitalizations in our region.

### Age as a factor for hospitalization

Our analysis identified age as a statistically significant independent predictor of hospitalization, with each additional year associated with a 4.7% increase in hospitalization risk. This outcome aligns with previous studies showing that older adults face a significantly increased risk of complications and hospital admissions due to influenza [21, 22]. Our data underscore the necessity of prioritizing older adults in preventative initiatives, owing to their biological vulnerability and the evident, measurable influence of age on severe disease results.

### Sex and influenza-related outcomes

In our research, sex did not significantly predict hospitalizations for patients exhibiting influenza-like illness. However, this finding differs from several earlier studies that suggested increased female vulnerability [23, 24]. Ludwig et al. [25] conducted a large cohort study of hospitalized patients in Germany, revealing that male patients exhibited a greater susceptibility to requiring intensive care, mechanical ventilation, and experiencing severe illness due to both COVID-19 and influenza.

The discrepancies may arise from variations in study design, populations, and endpoints. Some studies concentrate on outpatient symptom burden or lab-confirmed cases, whereas others, including ours, evaluate clinical severity through real-world emergency presentations. Additionally, biological mechanisms such as hormone-modulated immune responses, healthcare-seeking behavior, and baseline comorbidity profiles are likely to influence the observed sex disparities.

These sex-related findings should be interpreted with caution, as our cohort’s small number of hospitalized patients reduces statistical power to detect moderate associations. Larger, multicenter trials with larger samples are required to investigate potential sex-related variations in influenza outcomes.

### Comorbidities and hospitalization

Comorbidities are established predictors of negative outcomes in influenza infections. Research indicates that underlying conditions, including cardiovascular disorder, diabetes, chronic lung disease, and immunosuppression, are associated with heightened risks of hospitalization, mechanical ventilation, and mortality during influenza infection [26–28]. Our study found no statistically significant association between CCI and hospitalization. This result contrasts with existing literature, and several explanations are possible. Our sample size may have been insufficiently powered to identify the effects of comorbidity load. The possibility of underreporting or insufficient documentation of chronic diseases in acute care environments may have resulted in an underestimate of the actual comorbidity rates.

Consequently, these findings should be interpreted cautiously because of the small number of hospitalized patients and the low proportion of individuals with high comorbidity scores, which may have limited our ability to find statistically significant associations. Additionally, relying on administrative coding retrospectively and the variation in data quality among patient records may have introduced information bias. Future multicenter studies with larger sample sizes, standardized assessment of comorbidities, and rigorous validation of diagnostic codes are necessary to understand better the relationship between comorbidities and the risk of influenza-related hospitalization.

### Vaccination status and hospitalization

Our study indicated that influenza vaccination is linked with a significantly reduced risk of hospitalization, reflecting a 47% relative decrease in the likelihood of hospitalization among vaccinated individuals. This data corroborates extensive research illustrating the therapeutic benefits of influenza vaccination in mitigating disease severity and preventing complications [15, 29, 30].

The robust protective trend identified in our analysis reinforces established public health recommendations advocating for widespread annual influenza vaccination, particularly among high-risk populations. Our data contribute to this body of evidence by illustrating that, even within a relatively small cohort from the emergency department, vaccination status remains an independent and significant modifier of clinical outcomes. Considering the low vaccination rate observed in our population (3.3%), these findings highlight a crucial opportunity to enhance vaccine outreach and coverage to alleviate hospital burden during seasonal influenza peaks.

### Limitations

This study comprises several limitations that should be acknowledged when interpreting the results. The retrospective design presents a risk of selection bias and unmeasured confounding, especially when laboratory-confirmed influenza diagnoses are unavailable for all patients. Influenza diagnoses relied solely on ICD-10 codes in the electronic medical record system, without universal laboratory confirmation. This creates the possibility of misclassification bias, as coding practices might not perfectly match laboratory-confirmed cases. Nonetheless, this limitation is common in retrospective studies that utilize large-scale administrative or electronic health record data and should be considered when interpreting the results. Secondly, vaccination status was self-reported during follow-up, which may have introduced recall bias or misclassification, particularly among elderly patients or those with cognitive impairments. Another notable limitation of this study is the low vaccination rate. The restricted sample size of vaccinated individuals diminishes the statistical power to identify associations and may constrain the generalizability of the results. The total number of hospitalizations was limited. The reliance on single-center data restricts the external validity of our findings, as variations in healthcare-seeking habits, vaccination coverage, and admission boundaries across regions may influence generalizability. An additional restriction is the zero-event problem, which occurs when there are no hospitalizations among vaccinated patients, leading to undefined odds ratios in standard logistic regression. While Bayesian logistic regression was used to overcome this problem, the small number of vaccinated individuals may still bias the estimates, thus exaggerating the beneficial impact of vaccination. Therefore, these data should be regarded with caution.

## Conclusion

This retrospective, single-center investigation of patients with influenza-like illness presenting to the emergency department revealed that age was significantly associated with hospitalization, although sex and comorbidity burden were not independently linked to hospitalization risk. Notably, none of the vaccinated individuals necessitated hospitalization, and Bayesian logistic regression validated a statistically significant protective effect of seasonal influenza vaccination. Notwithstanding the restricted sample size and low vaccination rate, our findings augment the expanding corpus of evidence endorsing the effectiveness of immunization in alleviating severe consequences linked to influenza. Future studies should employ prospective, multicenter designs with larger and more diverse populations to further our understanding of the interplay among demographic, clinical, and behavioral factors that affect influenza outcomes. Such initiatives are crucial for generating generalizable evidence and informing targeted public health policies to mitigate the impact of seasonal influenza through effective vaccination outreach and risk stratification.

## Supplementary Information

Below is the link to the electronic supplementary material.

Supplementary Material 1 (PNG 47.6 KB)

Supplementary Material 2 (PNG 283 KB)

Supplementary Material 3 (DOCX 798 KB)

## Data Availability

Raw data can be provided to researchers upon request.
