# Supplementary figures and images for: Vaccination status as a determinant of hospitalization in influenza: Insights from emergency department data
Source: Eur J Clin Microbiol Infect Dis. 2026 Jan 8;45(4):1093–104. doi: 10.1007/s10096-025-05401-4 (PMC13086784; doi:10.1007/s10096-025-05401-4)

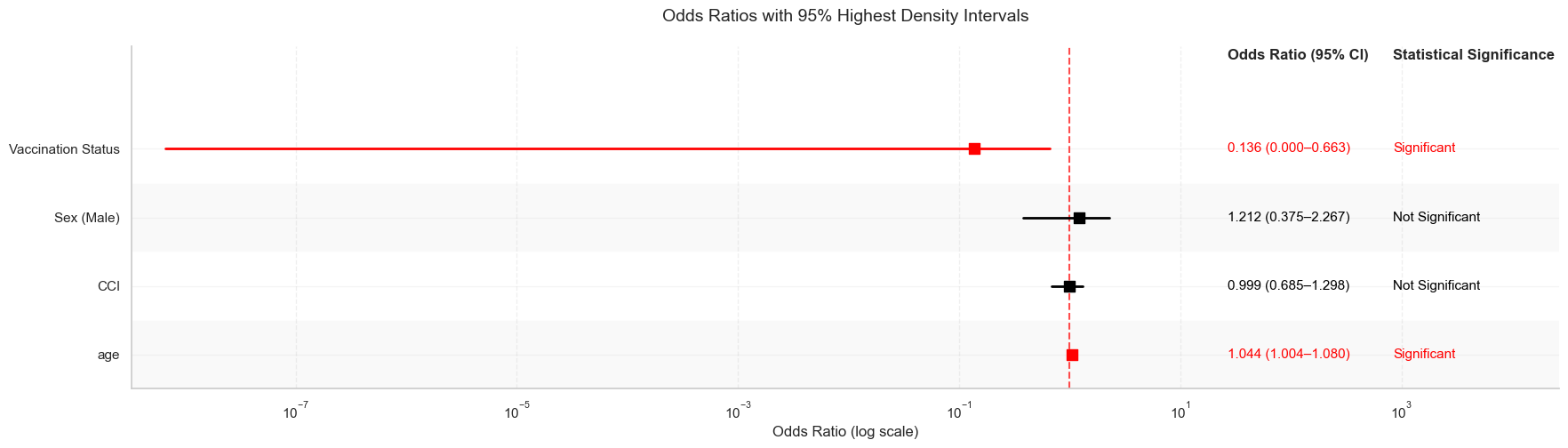

Supplement: Supplementary file 1 — Supplementary Material 1 (PNG 47.6 KB) [file 10096_2025_5401_MOESM1_ESM.png]

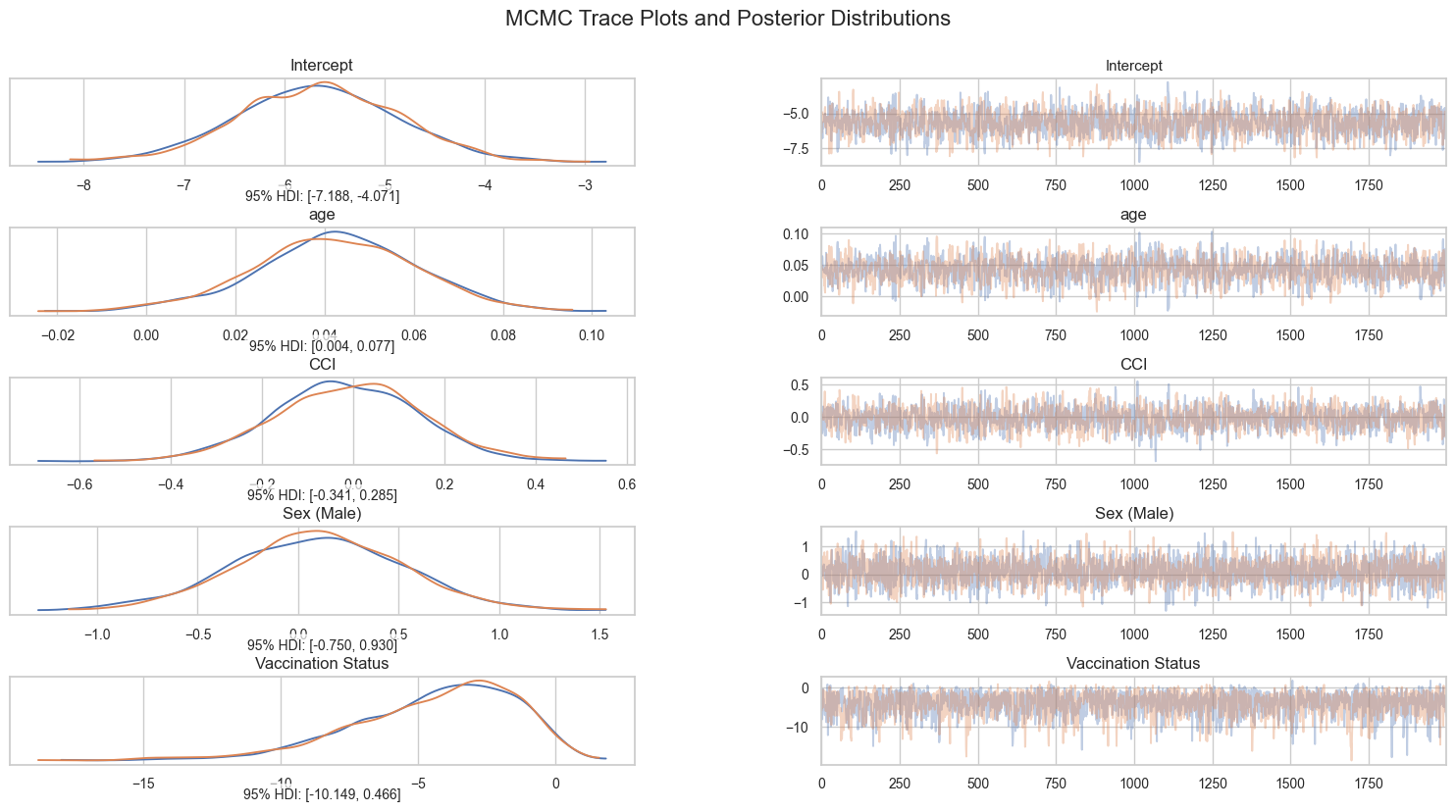

Supplement: Supplementary file 2 — Supplementary Material 2 (PNG 283 KB) [file 10096_2025_5401_MOESM2_ESM.png]

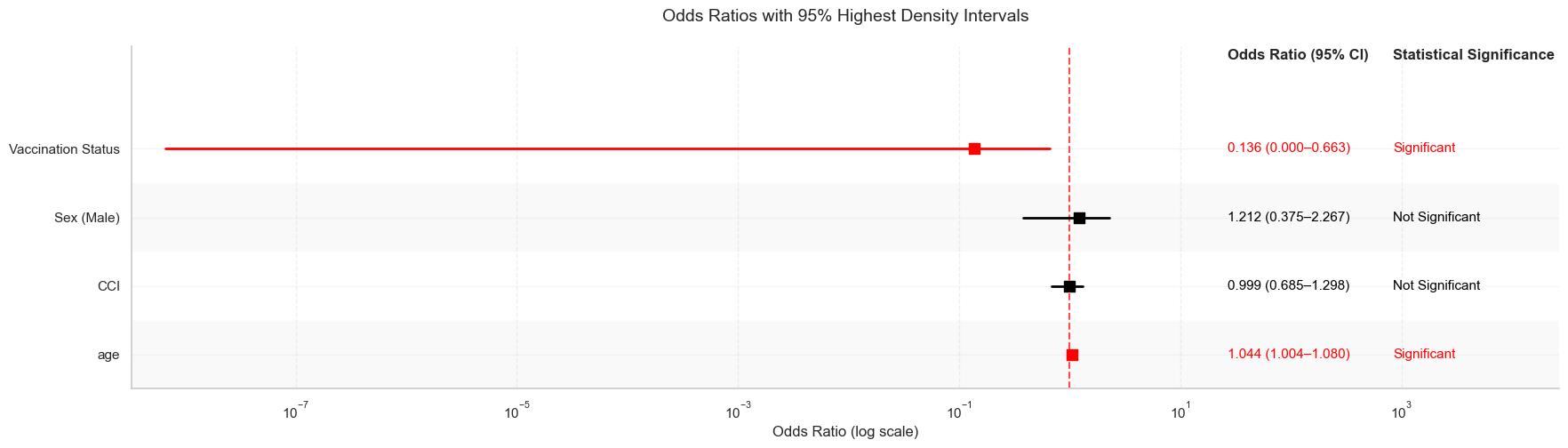


**bayesian_odds_ratio_plot**

**
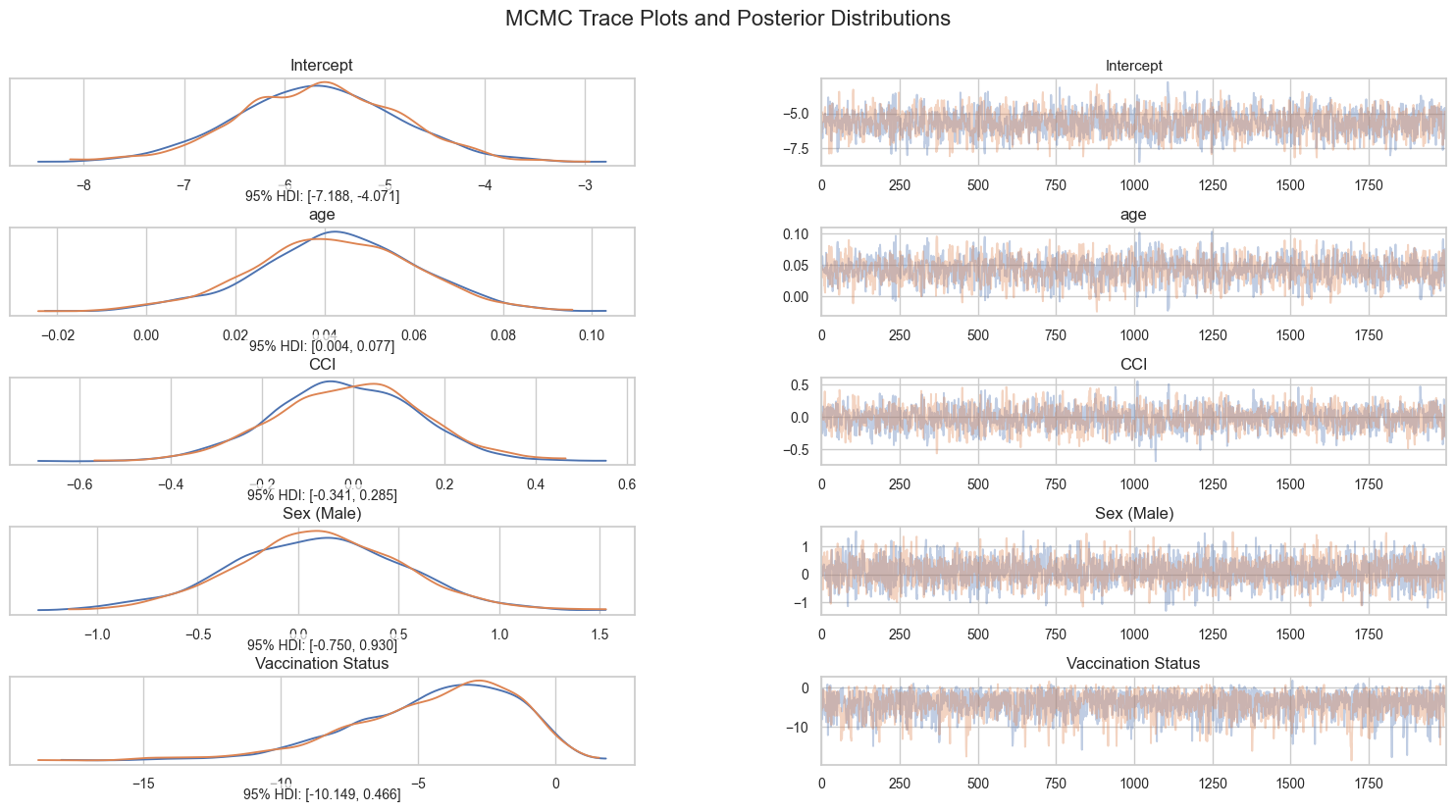
**

**bayesian_trace_plots_enhanced**

Supplement: Supplementary file 3 — Supplementary Material 3 (DOCX 798 KB) [file 10096_2025_5401_MOESM3_ESM.docx]
